# Topoisomerase II trapping agent teniposide induces apoptosis and G2/M or S phase arrest of oral squamous cell carcinoma

**DOI:** 10.1186/1477-7819-4-41

**Published:** 2006-07-06

**Authors:** Jinzhong Li, Wantao Chen, Ping Zhang, Ningyi Li

**Affiliations:** 1 Ninth People's Hospital, School of Medicine, Shanghai Jiao Tong University, Shanghai Key Laboratory of Stomatology, Shanghai, 200011, P. R. China; 2 Affiliated Hospital, School of Medicine, Qingdao University, Qingdao,266021, P.R.China

## Abstract

**Background:**

Teniposide (VM-26) has been widely used in the treatment of small cell lung cancer, malignant lymphoma, breast cancer, etc. However, there are few reports on VM-26 against oral cancers. The present study was designed to identify the effect of VM-26 against oral squamous cell carcinoma *in vitro*, and to provide evidence for the feasibility and effectiveness of VM-26 for application to the patients with oral cancer.

**Methods:**

Human tongue squamous cell carcinoma cell line, Tca8113, was used. Cells were incubated with different concentrations of VM-26 for a variety of time span. Cisplatin (CDDP) was employed as a control reagent. MTT assay was used to assess the inhibitory rate of Tca8113 growth. Flow cytometer (FCM), transmission electronic microscope (TEM) and fluorescence staining were employed for determining the cell apoptotic rate. Cell cycle distribution of Tca8113 incubated with VM-26 was examined by flow cytometer assay. Statistic software (SAS 6.12, USA) was used for one-way *ANOVA*.

**Results:**

The IC50 of VM-26 against Tca8113 cells was 0.35 mg/l and that of CDDP was 1.1 mg/l. The morphological changes of Tca8113 cells were observed with fluorescence microscope and TEM. Apoptotic morphological feature could be found in the nucleus. Apoptotic rate of Tca8113 cells incubated with 5.0 mg/l of VM-26 for 72 hours was 81.67% and cells waere arrested at S phase. However, when exposed to 0.15 mg/l of VM-26 for 72 hours, G2/M phase increased from 12.75% to 98.71%, while the apoptotic rate was 17.38%, which was lower than that exposed to 5.0 mg/l of VM-26.

**Conclusion:**

VM-26 could significantly induce apoptosis of oral squamous cell carcinoma and inhibit cell growth. There may be another pathway to induce apoptosis of oral squamous cell carcinoma cells except for G2/M phase arrest.

## Background

The 5-year survival rate was reported to reach 64.7% for patients with oral carcinomas, while that for patients at advanced stages was between 20.0% and 40.0% [1]. As one of three main therapies, chemotherapy may be a potential method to improve the survival rate and quality of life in patients with oral cancer, especially in advanced cancers. Cancers in oral cavity are different with cancers in other regions, which means cancers in this region are less chemosensitive, and there is still no special anticancer drug available, which is a hinder for further improvement of chemotherapeutic treatment against oral cancers. Topoisomerase II (Topo II) exists ubiquitously in cells and plays an essential role in DNA replication, transcription, chromosome formation and separation of sister chromatid [2-4]. Because of its essential role in cell growth, cell-cycle, as well as the high expression in proliferating cells, this enzyme is an ideal target for cancer chemotherapeutic drug [5,6]. Virtually every form of cancers that can be cured by systemic chemotherapy were always treated with regimens that focus on (or at least contain) drugs targeted to Topo II [7]. Indeed drugs targeted to Topo II are now indispensable components of systemic chemotherapy schedule which could effectively cure several known types of human cancers. Teniposide (4'-demethylepipodophyllotoxin thenylidene-beta-D-glucoside, VM-26) is a podophyllotoxin derivative and was found to be able to stabilize DNA-Topo II complex in DNA replication, thus damage the DNA and induce cellular apoptosis. It has been shown that in the treatment against small cell lung cancer, malignant lymphoma, breast cancer and ovarian cancer, etc through both clinical findings from chemotherapy results and *in vitro *chemosensitivity test of tumor specimen, anti-tumor effect on VM-26 was stronger than other chemotherapeutic drugs [8-10]. However, there were yet few reports on VM-26 against oral cancers. The purpose of this present study was to explore the apoptosis and concomitant cell cycle progression of Tca8113 cell treated with VM-26, and to provide evidence for the feasibility and effectiveness of VM-26 for application to oral cancers clinically.

## Methods

### Cell line

Tca8113 cell line derived from a patient with tongue squamous cell carcinoma, was used in this study and cultured in RPMI 1640 medium supplemented with 10% calf serum, 200,000 u/l penicillin and 200,000 u/l streptomycin. Cells were cultured in 25 ml culture flasks with 2 × 10^5 ^cells/ml, in humidified atmosphere with 5% CO_2 _at 37°C.

### Drugs and reagents

VM-26 (Bristol-Myers Squibb Co. USA, List: 3075–9), was dissolved in sterile double distilled water to a concentration of 1 g/l, and stored at -20°C. Cisplatin (CDDP, Qilu Co. China, List: 9908012) was dissolved to a final concentration of 0.1 g/l. 5.0 g/l MTT [3-(4,5-dimethylthiazol-2-yl)-2,5-diphenyl tetrazolium bromide] was purchased from Sigma Chemical-reagent Company. Annexin V-FITC Kit (Becton-Dickinson Co., USA) was used in this experiment.

### MTT assay

Logarithmically growing Tca8113 cells were trypsinized and made into single cell suspension then plated in 96-well culture plate at a concentration of 5 × 10^4 ^cells/well, eight columns for VM-26 and seven columns for CDDP in each plate, 3 wells in each column. After 24 hours of incubation, the medium of the 3 wells in each column were replaced with medium containing VM-26 of 0.15 mg/l, 0.5 mg/l, 1.5 mg/l, 5.0 mg/l, 15 mg/l and 45 mg/l or CDDP of 0.1 mg/l, 0.3 mg/l, 1.0 mg/l, 3.0 mg/l and 9.0 mg/l, respectively. Blank control wells were added medium without drugs. Cells were then cultured for another 24 hours, 48 hours, 72 hours, 96 hours and 120 hours. The supernatants were removed and 20 μl MTT solution was added in each well, followed with another 4 hours of culture. The supernatants were discarded carefully and 200 μl dimethyl sulphoxide (DMSO, Sigma, USA) was added and shaken vigorously to dissolve the purple precipitation formation. Optical density (OD) of each well was tested using Spectrophotometer (Bio-Tek instruments INC.) with a wavelength of 450 nm. The experiment was repeated in triplicate. The inhibitory rate (IR) of cell growth was calculated with the following formula:

IR = [(1-OD_t_)/OD_c_] × 100%

(ODt: OD of cells in treated groups; ODc: OD of cells in control groups).

### Cell fluorescence staining

Cells were incubated with VM-26 of 0.15 mg/l, 0.5 mg/l, 1.5 mg/l, 5.0 mg/l and 15 mg/l for 24 hours, 48 hours, 72 hours, 96 hours and 120 hours, respectively, then washed twice in phosphate buffered saline (PBS) and centrifuged at 1000 rpm for 10 minutes. Cell concentration was adjusted as 1 × 10^6 ^cells/ml. 10 μl of fluorescence dye EB/AO was added in 100 μl cell suspension, followed with 30 minutes of incubation in dark. Cells were then observed under fluorescence microscope. Nuclei of dead cells were dyed red while nuclei in live cells were dyed green.

### Observation of cell ultrastructure using TEM

Cells were cleansed using dimethylarsinic sodium, and fixed immediately with 2% glutaraldehyde for 30 minutes. The cells were then fixed with 1% osmium tetroxide for 2 hours at 4°C, dehydrated in ethanol of gradient concentrations, displaced twice using epoxy dimethylmethane, and permeated using 618 embedding solution. Ultrastructure of cell was observed and recorded using TEM Equipment (H-500, Japan).

### Cell apoptosis assay

The cells were incubated with VM-26 of 0.15 mg/l, 1.5 mg/l, 5.0 mg/l and 15 mg/l for 12 hours, 24 hours, 36 hours, 48 hours and 72 hours, respectively. Then the cells and medium supernatant were collected, washed twice with cold PBS, resuspended in 100 μl 1 × binding buffer (contained in Annexin V kit), stained with 5 μl Annexin V-FITC and 5 μl PI, and tested using flow cytometer (FACScalibur, Becton-Dickinson Co., USA).

### Cell-cycle assay

Tca8113 cells were treated with VM-26 of 0.15 mg/l, 1.5 mg/l, 5.0 mg/l and 15 mg/l for 12 hours, 24 hours, 36 hours, 48 hours and 72 hours, respectively. Then the cells were harvested, fixed in cold 70% ethanol for 30 minutes, and resuspended in 1 ml PBS. The cell suspensions were treated using 100 μl RNase of 1 g/l for 30 min, and stained with 100 μl PI of 50 mg/l in dark for 30 minutes, and then the cells were tested using FCM. The cell cycle distribution was analyzed using ModFit LT 3.0 software.

### Statistics

Data were analyzed with one-way *ANOVA *using SAS statistical software (6.12, TS20).

## Results

### Dose-dependent manner of VM-26 and CDDP against Tca8113 cells

**Figure 1:**
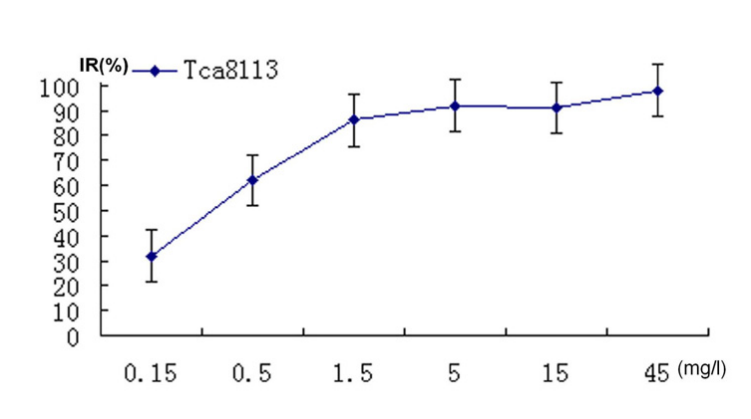
VM-26 inhibited the proliferation of Tca8113 cells in a dose-dependent way (cells were treated for 72 hours) according to the results of MTT assay. The inhibitory rates reached plateau when the dose of VM-26 increased to 1.5 mg/l. The IC50 of VM-26 was 0.35 mg/l. The figure was the representative of three independent experiments.

**Figure 2:**
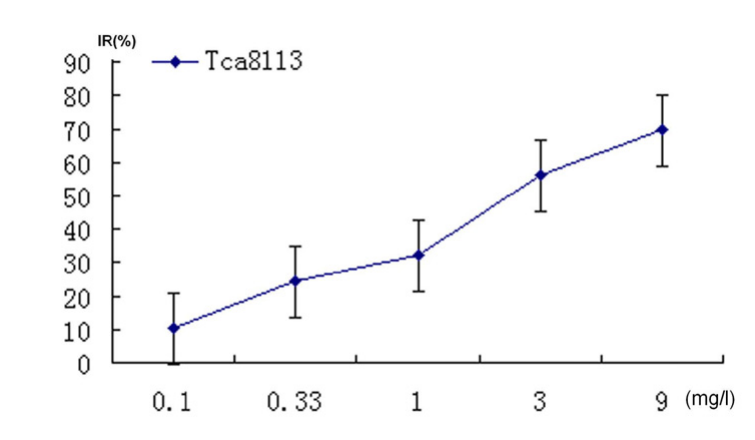
CDDP inhibited the proliferation of Tca8113 cells dose-dependently (cells were treated for 72 hours) according to the results of MTT assay. The IC50 of CDDP was 1.1 mg/l. The figure was the representative of three independent experiments.

0.5 mg/l of VM-26 showed significantly inhibitory effect in a dose-dependent manner. Cells shrank and round-shaped, accompanied with increasingly plasma vacuoles formation. Incubated with 0.5 mg/l of VM-26 (1/30 of average plasma concentration) for 72 hours, cell growth was inhibited significantly, and 83.58% Tca8113 cells were dead. IC50 of VM-26 was 0.35 mg/l (figure 1). However, 0.3 mg/l of CDDP (1/10 of average plasma concentration) could not inhibit cell growth significantly. The IC50 of CDDP was 1.1 mg/l (figure 2).

### Time-dependent manner of VM-26 and CDDP against Tca8113 cells

**Figure 3:**
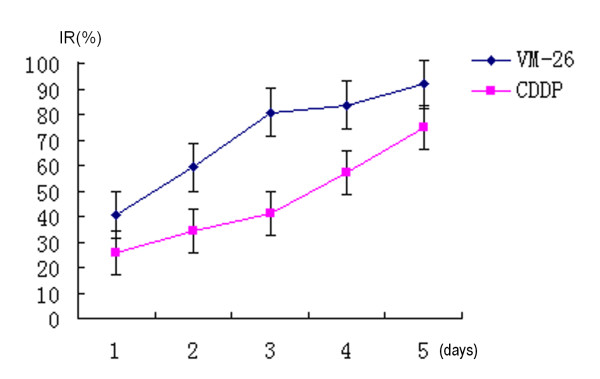
5.0 mg/l of VM-26 and 1.0 mg/l of CDDP inhibited the proliferation of Tca8113 cells time-dependently according to the results of MTT assay. VM-26 played potent inhibiting effect on the proliferation of Tca8113 cells compared with CDDP with the parallel plasma concentrations. The figure was the representative of three independent experiments.

Both VM-26 and CDDP showed growth inhibitory potency to Tca8113 cells in a time-dependent manner. VM-26 of 5.0 mg/l significantly inhibited the proliferation of Tca8113 cells. The inhibitory rate was 40.65% at 24 hours. As time went on, the inhibitory rate reached 80.9% at 72 hours and 92.1% at 96 hours. VM-26 had a stronger inhibitory potency than CDDP at the similar level of plasma concentration at any time span (figure 3).

### Apoptosis assay of Tca8113 cells

**Figure 4:**
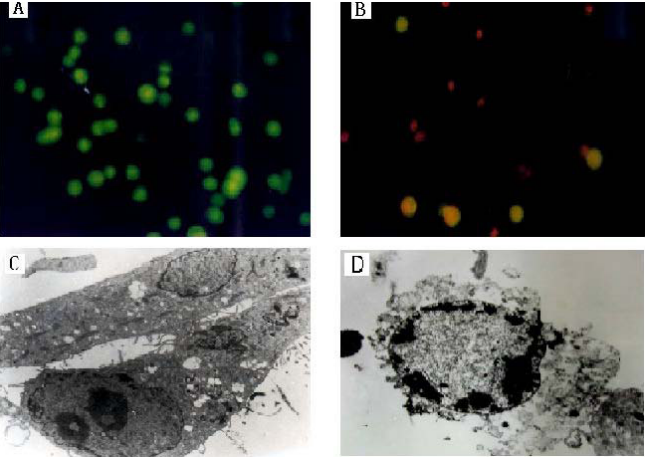
The morphological changes of Tca8113 cells treated with VM-26. Figure A showed the blank control cells were dyed green using EB/AO stained, figure B showed the cells exposed to 5.0 mg/l of VM-26 for 72 hours were dyed red. Figure C showed the feature of blank control cells, figure D showed the apoptotic phenomenon of Tca8113 cells exposed to 5.0 mg/l of VM-26 for 48 hours, chromosome condensed into a semilunar shape and clung to the karyotheca (TEM × 4000).

Fluorescence staining intuitively showed the inhibitory effect of VM-26. The nuclei of control Tca8113 cells stained with EB/AO dye were green (figure 4A). Treated with VM-26 of 0.5 mg/l for 24 hours, some nuclei became red and some dyed into yellow. With the concentration of VM-26 increasing or time lapsing, more cells became red, which confirmed the time-dependent and dose-dependent manner of VM-26 against Tca8113 cells. Figure 4B show the dead cells after incubation with 5.0 mg/l of VM-26 for 72 hours.

The ultrastructure changes during cell death were observed compared with the feature of blank control cells (figure 4C) using TEM. The cells treated with VM-26 showed typical apoptotic phenomenon. The nucleus became smaller, karyoplasm was concentrated and karyotheca was crimpled, chromosome condensed into a semilunar shape clinging to the karyotheca and the cellular membrane (figure 4D). The results indicated that Tca8113 cells were killed by VM-26 through apoptotic induction.

**Figure 5:**
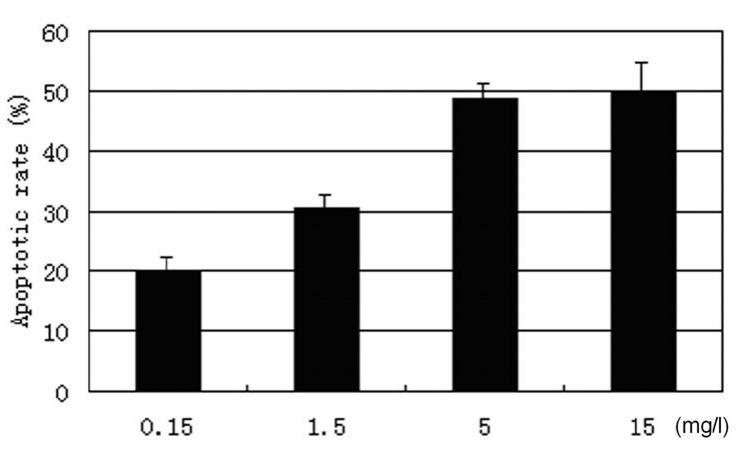
The apoptosis of Tca8113 cells were induced by VM-26 in a dose-dependent manner (for 48 hours) according to the results of Annexin-V/PI assay. 5.0 mg/l of VM-26 induced similar apoptotic rate as 15 mg/l of VM-26 did. The results were expressed as mean ± SD of three independent experiments.

**Figure 6:**
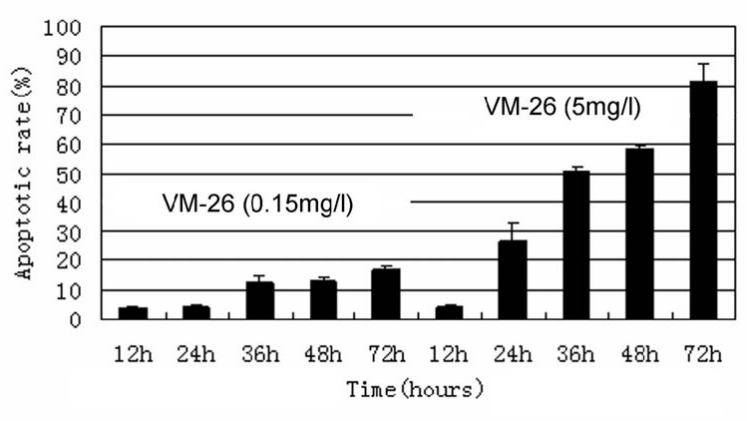
The apoptosis of Tca8113 cells were induced by VM-26 in a time-dependent manner according to the results of Annexin-V/PI assay. 5.0 mg/l of VM-26 demonstrated more apoptosis-inducing effect on Tca8113 cells than 0.15 mg/l did. The results were expressed as mean ± SD of three independent experiments.

Most Tca8113 cells were killed through apoptosis in a dose-dependent manner, which increased significantly with the increase of VM-26 concentration. Compared the 4 concentrations, 5.0 mg/l of VM-26 or 15 mg/l seemed to induce similar apoptosis in Tca8113 cells, which indicated that 5.0 mg/l of VM-26 was effective in inducing apoptosis (figure 5). Cells exposed to 0.15 mg/l of VM-26 seemed to just begin apoptosis at 36 hours, which approximate to population doubling time of Tca8113 cells (38.8 hours). However, cells treated with 5.0 mg/l of VM-26 began apoptosis at 24 hours and only 3.57% was viable after 72 hours (figure 6).

### Cell-cycle distribution

**Figure 7:**
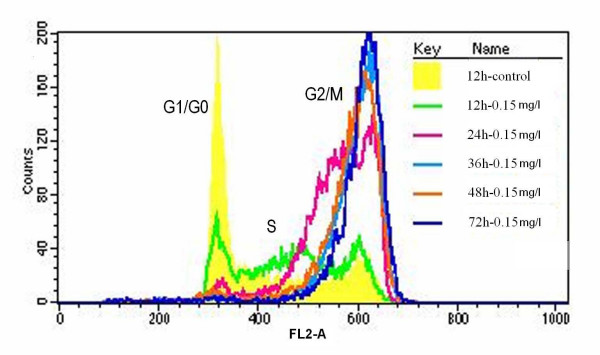
Overlay of cell cycle distributions of Tca8113 cells exposed to 0.15 mg/l of VM-26 for a variety of time span. The cells were harvested and analyzed with propidium iodide staining to assess for cell cycle distribution by FACS analysis. The line in yellow, green, red, light blue, brown and blue indicate the cell cycle distributions of control cells, and of cells treated with teniposide for 12 hours, 24 hours, 36 hours, 48 hours and 72 hours, respectively. The cell cycle of Tca8113 cells were obviously arrested at G2/M phase as time lapsed. The results were expressed as mean ± SD of three independent experiments.

**Figure 8:**
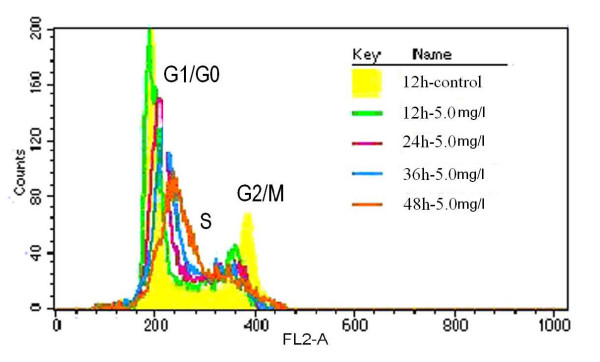
The cells were harvested and analyzed with propidium iodide staining to assess cell cycle distribution by FACS analysis and the results were expressed as mean ± SD of three independent experiments. Overlay of cell cycle distributions of Tca8113 cells exposed to 5.0 mg/l of VM-26. The line in yellow, green, red, light blue and brown indicated the cell cycle distributions of control cells, and of cells treated with VM-26 for 12 hours, 24 hours, 36 hours and 48 hours, respectively. The cell cycle of Tca8113 cells were obviously arrested at S phase as time lapsed.

Tca8113 cells exposed to VM-26 showed cell cycle arrest in a time-dependent manner. 98.71% of cells were arrested at G2/M phases after treated with 0.15 mg/l of VM-26 for 72 hours, (figure 7). Apoptosis of cells seemed to be induced through the G2/M phase arrest. While Tca8113 cells treated with 5.0 mg/l of VM-26 cell cycle was arrested mainly in S phase, and higher apoptotic rate occurred in this group (shown in figure 6 and figure 8). Cell cycle of Tca8113 cells exposed to 0.15 mg/l of VM-26 was arrested at G2/M phase, while in another group (containing 5.0 mg/l of VM-26) cells were arrested at S phase (figure 8).

## Discussion

Topo II has two isomers, TopoIIα and Topo IIβ. Topo IIα is cell-cycle regulated and is abundant in proliferating cells while Topo IIβ predominates in quiescent cells [11]. High expression of Topo IIα in tumor tissue is a common phenomenon, and Topo IIα is gradually regarded as a sign to judge the extent of tumor cell proliferating [12-14]. Topo II poisons can be classified as DNA-intercalating drug and DNA-nonintercalating drug. The former can damage DNA through inserting the plane portion of its molecular structure, such as the ring of purine or pyrimidine, into the double strands of DNA where Topo II binding to interfere the enzyme religation of the nicks of the broken DNA double strands, which lead cell to death eventually. However, the mechanism of non-intercalating drug killing tumor cells is still unclear. VM-26 is a kind of non-intercalating TopoII poison. Generally speaking, drug that can inhibit Topo IIα is also capable of inhibiting Topo IIβ. However, most drugs, especially non-intercalating drugs, are more effective on Topo IIα while intercalating drugs are more effective on Topo IIβ [15]. Chen *et al *[16] applied 8 drugs (CDDP, 5-Fu, PYM, Taxol, VM-26, E-ADM, VDS and MTX), to test the chemosensitivity of 140 oral cancer specimens before chemotherapy, finding the average inhibitory rate of VM-26 against tumor cells reached 63.76% while the inhibitory rates of other 7 drugs were less than 30.0%. These oral squamous cell carcinoma specimens seemed not sensitive to E-ADM which is an intercalating Topo II poison.

CDDP was used as a positive control drug because it was in wide clinical use and regarded as a standard chemotherapeutic drug. We chose experimental concentration of these two drugs according to their average plasma concentration because it determined their clinical effects. Both the MTT and the FCM assay indicated that, at 5.0 mg/l concentration, the potency of VM-26 inhibiting Topo II and inducing apoptosis reached plateau. In any case, 5.0 mg/l (1/3 average plasma concentration [17]) VM-26 could induce approximate maximum inhibitory effect on Tca8113 cells *in vitro*. We found that 5.0 mg/l of VM-26 could inhibit oral cancer cells significantly after 72 hours incubation; the inhibitory rate reached 92.1% at 120 hours, which was much higher than those of CDDP at the 1/3 average plasma concentration. VM-26 inhibits the growth of tumor cell in an obvious dose-dependent and time-dependent manner.

The typical apoptotic phenomenon was found in Tca8113 cells treated with VM-26 by TEM, and also, apoptosis were induced by VM-26 both dose-dependently and time-dependently. There are many pathways for cells apoptosis, including PKA/camp pathway, T cell receptor pathway, dead receptor pathway and Fas/TNFR1 associated pathway. Mo *et al *[4] found that the ability of these DNA Topo II inhibitors including VM-26 activating FasL promoter and inducing FasL expression, was correlated with their ability to cause DNA damage. When these permanent DNA breaks were present in sufficient numbers, they initiated a series of apoptosis events that ultimately culminated in cell death [7].

G2 phase arrest was a common phenomenon found in mammalian cells when they were treated with most DNA-damaging reagents [18]. Such G2 arrest has been proposed to be due to induction of G2 checkpoint machinery that allows damaged DNA to be repaired before cells move to the next cell cycle stage [19,20]. Chen *et al *[21] reported that cells treated with VM-26 of higher dosage stopped the cell cycle in S phase, but they found the cells at last be arrested in the G2/M phase if the time of incubation with drug was prolonged and assumed such S phase inhibition should be a kind of S phase retardation other than arrest. The present study showed only VM-26 of 0.15 mg/l could significantly arrest cells in G2/M phase, while VM-26 of 1.5 mg/l, 5.0 mg/l and 15 mg/l tended to stop cell progression in S phase, and initiated significant apoptosis concomitantly. Thus, this kind of apoptosis was not triggered by G2 checkpoint machinery. This study found apoptosis of cells incubated with VM-26 of 5.0 mg/l was much more than that of cells with VM-26 of 0.15 mg/l, that is, much more cells died in the S phase than cells did in G2/M phase. So, apoptosis of Tca8113 cells incubated with VM-26 might not be induced by G2/M phase arrest, apoptosis could be induced before cells reached G2 checkpoint. Cells do not always get into G2/M phase when treated with VM-26. There may be another pathway to induce apoptosis of oral carcinoma cells except traditional G2/M phase arrest.

## Conclusion

The present study showed that Tca8113 cells were sensitive to VM-26 and provided evidences for applying VM-26 in oral cancer chemotherapy. The results also showed that low dose and high dose of VM-26 worked in different way and there existed other ways except for G2/M phase arrest.

## Competing interests

The author(s) declare that they have no competing interests.

## Authors' contributions

JL carried out cell culture, cell apoptosis assay, cell cycle assay, performed statistical analysis and drafted the manuscript. WC conceived of the study, carried out MTT assay, cell fluorescence staining and TEM observation, and drafted the manuscript.. PZ helped to draft the manuscript. NL participated in its design and coordination. All authors read and approved the final manuscript.
